# Achievement of visible-light-driven Z-scheme overall water splitting using barium-modified Ta_3_N_5_ as a H_2_-evolving photocatalyst†
†Electronic supplementary information (ESI) available. See DOI: 10.1039/c6sc02750d



**DOI:** 10.1039/c6sc02750d

**Published:** 2016-08-18

**Authors:** Yu Qi, Shanshan Chen, Mingrun Li, Qian Ding, Zheng Li, Junyan Cui, Beibei Dong, Fuxiang Zhang, Can Li

**Affiliations:** a State Key Laboratory of Catalysis , iChEM , Dalian Institute of Chemical Physics , Chinese Academy of Sciences , Dalian National Laboratory for Clean Energy , Dalian , 116023 , China . Email: fxzhang@dicp.ac.cn ; Email: canli@dicp.ac.cn ; http://canli.dicp.ac.cn; b University of Chinese Academy of Sciences , Beijing 100049 , China; c Key Laboratory of Surface and Interface Chemistry of Jilin Province , College of Chemistry , Jilin University , Changchun 130021 , China

## Abstract

Ta_3_N_5_ is one of the most promising photocatalyst candidates for solar water splitting, but it still remains challenging to achieve overall water splitting *via* Ta_3_N_5_-based photocatalysts regardless of whether it uses a one step or two step method. Here we will address the relatively poor photocatalytic proton reduction of Ta_3_N_5_ with an effort for the promotion of charge separation *via* barium modification. One-pot nitridation of barium nitrate-impregnated Ta_2_O_5_ precursor was adopted here for the synthesis of Ta_3_N_5_ accompanied with the creation of a Ta_3_N_5_/BaTaO_2_N heterostructure and surface passivation. Due to the synergetic effect of the improved interfacial charge separation and the decreased defect density, the photocatalytic H_2_ evolution rate of barium-modified Ta_3_N_5_ is effectively promoted. Encouraged by this, a visible-light-driven Z-scheme overall water splitting system was successfully constructed by using the barium-modified Ta_3_N_5_ as a H_2_-evolving photocatalyst, together with a PtO_*x*_/WO_3_ and IO_3_^–^/I^–^ pair as an O_2_-evolving photocatalyst and a redox mediator, respectively.

## Abstract 2

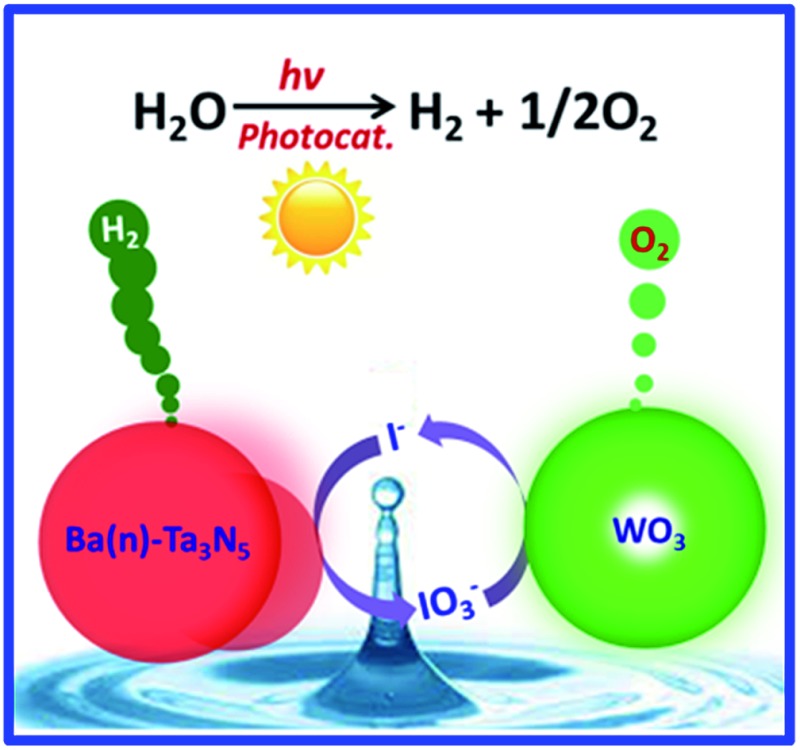
Barium-modified Ta_3_N_5_ for the promotion of proton reduction is first employed as a H_2_-evolving photocatalyst for visible-light-driven Z-scheme overall water splitting.

## Introduction

Semiconductor-based photocatalytic overall water splitting for hydrogen production is an ideal way to convert solar energy to chemical energy and has inspired extensive interest in the past few decades.^1–5^ Towards this, hundreds of semiconductors have been reported for potential solar water splitting, but most of them are only active under UV light irradiation.^6–10^ To achieve highly efficient solar-to-chemical energy conversion, overall water splitting on photocatalysts harvesting visible light with longer wavelength is desirable. To date, however, the number of wide visible-light-driven overall water splitting systems, regardless of whether they use a one step or two step method, is limited.^5,11–18^

Tantalum nitride (Ta_3_N_5_), with a theoretical solar-to-hydrogen conversion efficiency of 15.9%, is one of the most promising candidates for solar water splitting, considering its matched band edge positions (conduction band and valence band edges at *ca.* –0.4 V and +1.7 V *vs.* NHE, respectively, at pH = 0), wide visible light harvesting ability (up to 600 nm) and good photo-stability.^19–37^ It was first synthesized in 1973,^38^ but was not found to be active for the photocatalytic water splitting reaction until 2002.^19^ Afterwards, Ta_3_N_5_ has been widely investigated for water splitting in terms of particulate photocatalysts^22–25^ and photoanodes.^26–31^

The increasing research interest and efforts have greatly promoted the water oxidation performance of Ta_3_N_5_ for both particulate photocatalyst and photoanode systems. For example, Li *et al.* fabricated a 1D Ta_3_N_5_ nanorod photoanode to achieve a STH of 1.5%.^30^ Liu *et al.* achieved Ta_3_N_5_ photoanode stability for hours^27^ and obtained nearly close to the theoretical photocurrent at a potential of 1.23 V *vs.* RHE under AM 1.5G simulated sunlight.^31^ Chen *et al.* reported that the apparent quantum efficiency of the photocatalytic water oxidation activity of the Ta_3_N_5_-based particulate photocatalyst can reach 11.3% at 500–600 nm *via* an interface engineering strategy.^24^ Compared to the water oxidation, however, the activity of photocatalytic proton reduction from water is much lower or even undetectable in most cases, even though extensive investigations such as forming polymorphic macroporous Ta_3_N_5_, reducing the particle size through templates (*i.e.* SiO_2_, C_3_N_4_) and surface modification have been made.^32–37^ As a result of the poor proton reduction ability, Z-scheme overall water splitting using particulate Ta_3_N_5_ as a H_2_-evolving photocatalyst is still not reported.

Fabricating nanocomposites with another semiconductor to form heterostructures has been extensively adopted for the promotion of photocatalytic performances.^16,39–42^ A heterostructure can create external bias through interfacial junctions to spatially separate the photogenerated electrons and holes. However, it should be pointed out that most of the (oxy)nitride photocatalysts are thermally instable in air, so the fabrication of a heterostructure for (oxy)nitride commonly confronts technical challenges, rendering feasible examples very limited.^16^

In this work, a barium modification strategy is introduced to address the relatively poor photocatalytic proton reduction activity of Ta_3_N_5_ under visible light irradiation. A simple one-pot nitridation route was adopted for the synthesis of pristine Ta_3_N_5_ and barium-modified Ta_3_N_5_, in which a barium nitrate-impregnated Ta_2_O_5_ was used as a precursor. It is found that some Ba^2+^ ions could be doped into Ta_3_N_5_ to decrease its defect density. On the other hand, excessive Ba^2+^ ions will produce BaTaO_2_N *in situ* on the surface of Ta_3_N_5_ to create a Ta_3_N_5_/BaTaO_2_N heterostructure. As a result, the photogenerated carrier separation efficiency of Ta_3_N_5_ can be promoted after the barium modification, causing an effectively enhanced H_2_ evolution rate in the presence of methanol. Finally, the first example of a visible-light-driven photocatalytic Z-scheme overall water splitting system using the modified Ta_3_N_5_ as a H_2_-evolving photocatalyst was successfully constructed.

## Experimental

### Materials and reagents

For the preparation of Ba(*n*)-Ta_3_N_5_ samples, Ta_2_O_5_ (99.99%, Amresco Chemical), and Ba(NO_3_)_2_ (99.5%, Sinopharm Chemical) were used. WO_3_ (99.99%, High Purity Chemical) was used as a water oxidation photocatalyst. H_2_PtCl_6_·6H_2_O (99.5%, Sinopharm Chemical) was employed as the precursor for the reduction cocatalyst. CH_3_OH (99.5%, Sinopharm Chemical) and NaI (99.5%, Guangfu Chemical) were used as sacrificial electron donors. La_2_O_3_ (99.95%, Sinopharm Chemical) was applied as a pH buffer agent. All chemicals were used as purchased without further purification.

### Preparation of Ba(*n*)–Ta_3_N_5_ samples

Typically, Ta_2_O_5_ powder was impregnated in the Ba(NO_3_)_2_ aqueous solution with a calculated molar ratio of Ba/Ta, and the dried mixture was then annealed in air at 1073 K for 2 h. The as-prepared powder was treated with “one-pot” nitridation under ammonia flow (250 mL min^–1^) at 1223 K for 20 h. The as-obtained samples are correspondingly denoted as Ba(*n*)–Ta_3_N_5_, where “*n*” stands for the molar ratio of Ba/Ta and when *n* = 1 it stands for BaTaO_2_N. As a comparison, the pure phase of Ta_3_N_5_ and BaTaO_2_N powder was mechanically mixed at a Ba/Ta molar ratio of 0.3, which is denoted as Ta_3_N_5_/BaTaO_2_N (0.3)-mix.

### Deposition of cocatalysts

0.2 g of the as-obtained sample was dispersed in a calculated amount of H_2_PtCl_6_ aqueous solution, and sonicated for *ca.* 5 min. After the solution was completely evaporated in a water bath at 353 K, the resulting powder was collected and reduced at 473 K for 1 h under a flow of 5% H_2_/Ar (200 mL min^–1^). As for the deposition of PtO_*x*_ on the surface of WO_3_ for water oxidation, typically, 0.3 g of WO_3_ was annealed in the air at 773 K for 2 h, and then 0.2 g of the annealed sample was immersed in a calculated amount of H_2_PtCl_6_ aqueous solution with sonicating for *ca.* 5 min. After complete evaporation in a water bath at 353 K, the resulting powder was collected and annealed in air at 798 K for 0.5 h.

### Electrochemical analysis

For the Mott–Schottky (M–S) measurement, Ta_3_N_5_ and BaTaO_2_N powder were deposited on FTO conducting glass *via* electrophoretic deposition (EPD). Typically, the powder samples (50 mg) and iodine (20 mg) were dispersed in acetone solution (50 mL), and continuously sonicated for 10 min. Afterwards, the FTO electrode was immersed, parallel to another FTO electrode, with a distance of about 1 cm. The duration time was 1 min with 20 V and 1 A applied using a potentiostat (ITECH IT6834), and then the prepared electrodes were calcined under an ammonia flow (250 mL min^–1^) at 723 K for 0.5 h.

The M–S measurement was carried out using a Princeton Applied Research PARSTAT 2273, using 0.5 M Na_2_SO_4_ aqueous solution as electrolyte with a pH value of 8.5 adjusted using NaOH. The frequency was 1 kHz.

### Characterizations of catalysts

XRD measurements were carried out using a Rigaku D/Max-2500/PC powder diffractometer (Cu Kα radiation) with an operating voltage of 40 kV and an operating current of 200 mA. A scan rate of 5° min^–1^ was applied in the range of 10–60°. UV-vis diffuse reflectance spectra (DRS) were recorded using a UV-vis spectrophotometer (JASCO V-550) equipped with an integrating sphere, and BaSO_4_ powder was used as the reference for the baseline correction. The morphologies and particle sizes were examined using field emission scanning electron microscopy (FESEM; S-5500, Hitachi). High-resolution transmission electron microscopy (HRTEM) images were obtained using a Tecnai G2 F30 S-Twin (FEI Company) with an accelerating voltage of 300 kV. For the time-resolved IR spectroscopic study, the photocatalyst was fixed on a CaF_2_ plate at a density of 2 mg cm^–2^ and placed in a gas cell evacuated at 10^–5^ Torr. The Brunauer–Emmett–Teller (BET) surface area was measured at 77 K using a Micromeritics ASAP 2000 adsorption analyzer. Transient IR absorption signals were recorded on a Nicolet 870 FTIR spectrometer with a MCT detector. A pulse laser at 355 nm (1 Hz, 3 mJ per pulse) was used to excite the samples. The width of the laser pulse was 6–8 ns and no deconvolution on the data was carried out.

### Photocatalytic reactions

Photocatalytic reactions were carried out in a Pyrex top-irradiation type reaction vessel connected to a closed gas circulation system. Before photoirradiation, the reaction system was evacuated to completely remove air, and then irradiated from the top side using a 300 W xenon lamp with a filtration mirror equipped with an optical filter (Hoya, L-42; *λ* > 420 nm) to cut off the ultraviolet light. A flow of cooling water was used to keep the reaction suspension at room temperature. Gas chromatography (Agilent; GC-7890A, MS-5A column, TCD, Ar carrier) was used to analyze the evolved gases. The pH value before and after the photocatalytic overall water splitting reaction was similarly kept at *ca.* 6.

### Measurement of AQE

The AQE measurement was carried out using a Pyrex top-irradiation-type reaction vessel and a 300 W xenon lamp fitted with a 420 nm band-pass filter. The number of photons reaching the reaction solution was measured using a calibrated Si photodiode (LS-100, EKO Instruments Co., LTD.), and the AQE (*φ*) was calculated according to the following equation:*φ*(%) = (*AR*/*I*) × 100where *A* represents a coefficient (4 for H_2_ evolution; 8 for O_2_ evolution), *R* represents the evolution rate of H_2_ or O_2_ in the initial one hour irradiation and *I* represents the absorption rate of incident photons. It was assumed that all incident photons were absorbed by the suspension. The total number of incident photons at a wavelength of 420 nm was measured to be 4.76 × 10^20^ photons per h.

## Results and discussion

**Fig. 1:**
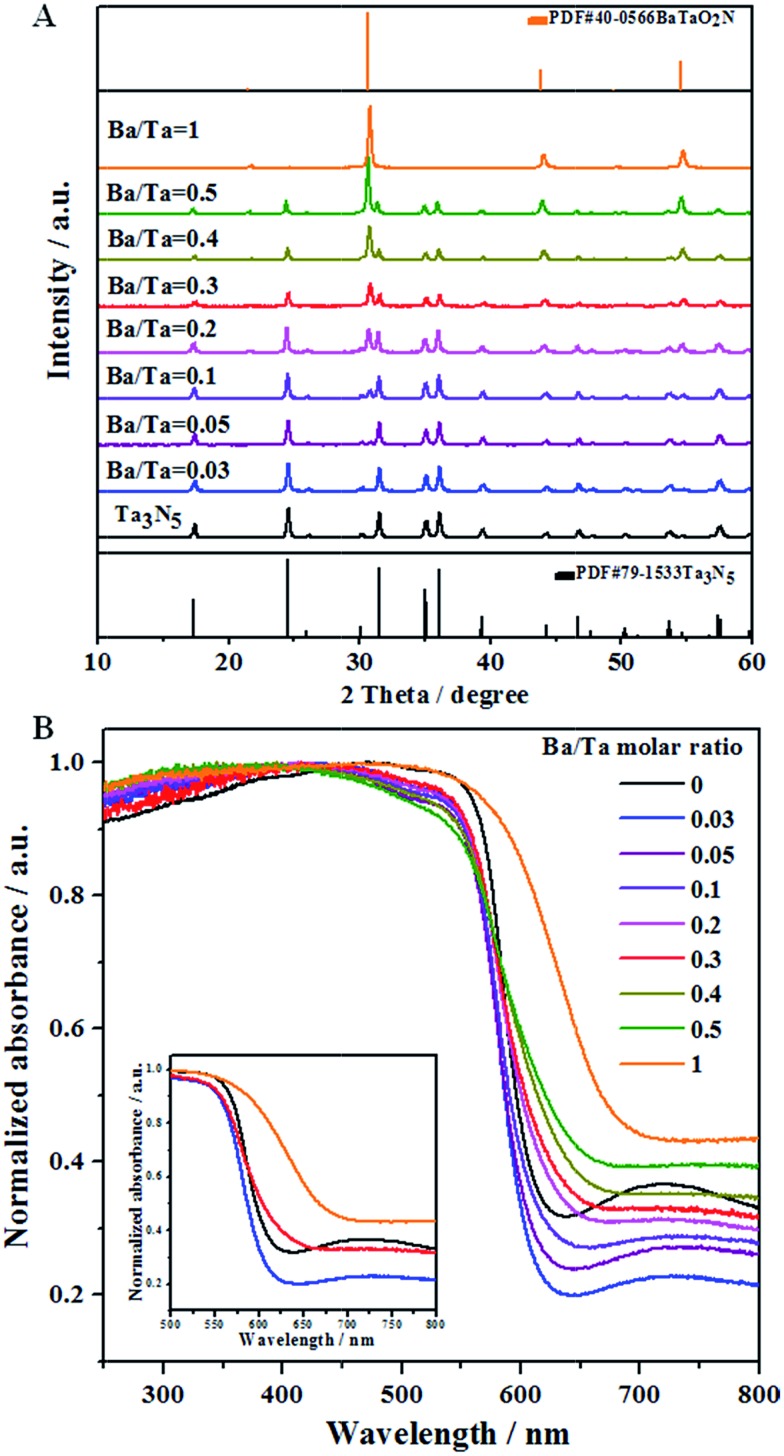
Structural characterizations of typical Ba(*n*)–Ta_3_N_5_ samples (*n* = 0–1): (A) XRD patterns and (B) UV-vis spectra. “*n*” stands for the molar ratio of Ba/Ta. The inset figure is enlarged for the wavelength range of 500–800 nm.

Fig. 1A shows XRD patterns of the Ba(*n*)–Ta_3_N_5_ samples (*n* = 0–1), in which all of them exhibit a well-crystallized feature. When the Ba/Ta molar ratio is below 0.03, only diffraction peaks assigned to a single phase of Ta_3_N_5_ are observed. With a further enhanced molar ratio of Ba/Ta, additional diffraction peaks attributed to BaTaO_2_N appear, the intensities of which are continuously increased. Compared with the diffraction peaks of the unmodified Ta_3_N_5_ sample, a little shift in the diffraction peaks toward a lower angle is observed for the barium-modified Ta_3_N_5_ samples (Fig. S1†). This demonstrates that the six-coordinated Ba^2+^ may be partly doped into Ta_3_N_5_ to substitute the Ta^5+^ sites, similar to the previous report.^30^

Fig. 1B shows the UV-vis spectra of the Ba(*n*)–Ta_3_N_5_ samples, in which all of the samples similarly exhibit a wide visible light absorption at around 600 nm. The absorption edge is continuously red-shifted with the increasing molar ratio of Ba/Ta, which should be the result of the formed BaTaO_2_N species. Compared to the pristine Ta_3_N_5_ sample, the absorption background originating from the formation of reduced tantalum species (*e.g.*, Ta^4+^ and Ta^3+^)^43,44^ on the Ba(*n*)–Ta_3_N_5_ samples undergoes an initial decrease and a subsequent increase with the enhancing molar ratio of Ba/Ta. To understand the UV-Vis results, a single phase of BaTaO_2_N was prepared *via* the same preparation procedure. As shown in Fig. 1B, the absorption edge of BaTaO_2_N is at about 660 nm, and its absorption background is the highest among all of the Ta_3_N_5_-based samples. It is generally understood that the UV-vis absorption background of the mechanically mixed sample containing two phases should be located between that of the corresponding single phases. That is to say, if the Ba(*n*)–Ta_3_N_5_ samples are just a simple mixture of Ta_3_N_5_ and BaTaO_2_N, the absorption background of the Ba(*n*)–Ta_3_N_5_ samples will lie between those of the Ta_3_N_5_ and BaTaO_2_N. In this work, however, the samples with a molar ratio of Ba/Ta below 0.3 exhibit much lower absorption backgrounds than those of both Ta_3_N_5_ and BaTaO_2_N. This means that the as obtained Ba(*n*)–Ta_3_N_5_ samples do not exist as a simple mixture of Ta_3_N_5_ and BaTaO_2_N, but exist as a nanocomposite. In consideration of that, here the Ta_3_N_5_ and BaTaO_2_N phases are one-pot synthesized, thus the BaTaO_2_N is expected to be formed *in situ* on the surface of Ta_3_N_5_ to partly eliminate the surface dangling bonds of Ta_3_N_5_. On the other hand, the partial barium ions are doped into Ta_3_N_5_ to inhibit the defect formation. Both of them cause the decrease of defect density. However, it needs to be pointed out that BaTaO_2_N itself exhibits the highest defect density among all of the samples. Thus, when the molar ratio of Ba/Ta is excessively enhanced, the defect density of the Ba(*n*)–Ta_3_N_5_ sample will become higher than that of Ta_3_N_5_. All of these factors should be integrally responsible for the initial decrease and subsequent increase of the absorption background (*i.e.* defect density) with the increasing molar ratio of Ba/Ta in the UV-Vis results (Fig. 1B).

**Fig. 2:**
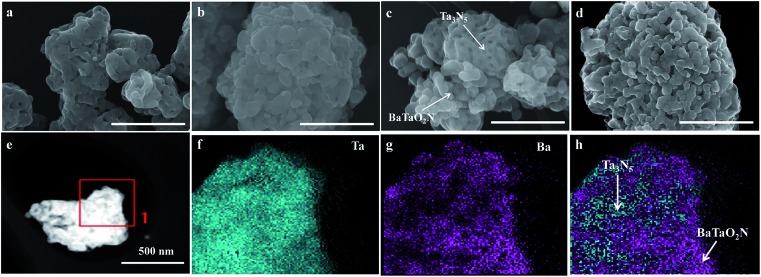
FESEM images of typical samples: (a) Ta_3_N_5_, (b) BaTaO_2_N, (c) a mixture of Ta_3_N_5_ and BaTaO_2_N, and (d) Ba(0.3)–Ta_3_N_5_ (the scale bar is 500 nm). Elemental mappings of Ba(0.3)–Ta_3_N_5_: (e) TEM image, (f) Ta element, (g) Ba element, and (h) simulated dispersion of Ta_3_N_5_ and BaTaO_2_N.

Fig. 2 shows FESEM images of typical samples. The Ta_3_N_5_ sample is porous (Fig. 2a), while the BaTaO_2_N sample has a shortage of porosity (Fig. 2b). The difference in their morphology can be easily judged from their mixed sample (Fig. 2c). However, the morphology feature of the chosen Ba(0.3)–Ta_3_N_5_ sample (Fig. 2d) prepared in this work is quite different from those of the corresponding single phases (Fig. 2a and b) or their mixed sample (Fig. 2c). As can be seen in Fig. 2d, the Ba(0.3)–Ta_3_N_5_ sample exhibits a homogeneous morphology with the two phases difficult to distinguish, demonstrating their interaction with each other as a nanocomposite. The formation of the Ta_3_N_5_/BaTaO_2_N nanocomposite can be further supported by the elemental mapping results (Fig. 2e–h). In Fig. 2f, the Ta element originating from both Ta_3_N_5_ and BaTaO_2_N is dispersed everywhere, while the Ba element that can only result from BaTaO_2_N is only found in some specific places (Fig. 2g). This can be easily understood to show that the places with Ba element mapping mainly reveal the existence of BaTaO_2_N, while the locations with Ta element mapping but a shortage of Ba element mapping stand for the Ta_3_N_5_ species. Based on the elemental mapping images, we can reasonably give a simulation of the composite state of Ta_3_N_5_ and BaTaO_2_N for the Ba(0.3)–Ta_3_N_5_ sample (Fig. 2h). For comparison, the element mapping results of mechanically mixed Ta_3_N_5_ and BaTaO_2_N (Ta_3_N_5_/BaTaO_2_N (0.3)-mix) are given in Fig. S2,† from which the Ta_3_N_5_ and BaTaO_2_N phases are mainly separated, different from that of the Ba(0.3)–Ta_3_N_5_ sample. It needs to be pointed out that the composite of Ta_3_N_5_ and BaTaO_2_N does not exist in a core–shell configuration. In addition, the surface areas of the Ba(*n*)–Ta_3_N_5_ samples are similar to that of BaTaO_2_N (7 m^2^ g^–1^) but a little lower than that of Ta_3_N_5_ (9 m^2^ g^–1^), which should result from their shortage of porous structure (Table 1).

**Table 1:** Photocatalytic performances of typical photocatalysts under visible light irradiation (*λ* > 420 nm)

Entry	H_2_-evolving photocatalysts	Surface area (m^2^ g^–1^)	Half reaction^*a*^	Overall water splitting^*b*^	
			H_2_ evolution rate (μmol h^–1^)	Gas evolution rates (μmol h^–1^)	
				H_2_	O_2_
1	Ba(0)–Ta_3_N_5_	9	0.05	0	0
2	Ba(0.03)–Ta_3_N_5_	7	0.1	Trace	Trace
3	Ba(0.05)–Ta_3_N_5_	7	4.2	0.8	0.4
4	Ba(0.1)–Ta_3_N_5_	7	6.6	2.0	1.0
5	Ba(0.2)–Ta_3_N_5_	7	19.3	2.5	1.3
6	Ba(0.3)–Ta_3_N_5_	7	30.2	3.2	1.6
7	Ba(0.4)–Ta_3_N_5_	7	28.2	3.0	1.5
8	Ba(0.5)–Ta_3_N_5_	7	24.6	2.1	1.1
9	BaTaO_2_N	7	9.5	0.3	0.15
10	Ta_3_N_5_/BaTaO_2_N (0.3)-mix	8	16.5	0.6	0.3

^*a*^Reaction conditions: 0.15 g of 0.5 wt% Pt/Ba(*n*)–Ta_3_N_5_ (*n* = 0–1) and 0.5 wt% Pt/Ta_3_N_5_/BaTaO_2_N (0.3)-mix samples; 0.15 g of La_2_O_3_; aqueous methanol solution (150 mL, 20 vol%); 300 W xenon lamp (*λ* > 420 nm); 1 h irradiation.

^*b*^Reaction conditions: 50 mg of 0.5 wt% Pt-modified H_2_-evolving photocatalysts; 50 mg of 0.45 wt% PtO_*x*_/WO_3_ as O_2_-evolving photocatalyst; 100 mL of aqueous NaI solution (1.0 mM); Pyrex top-irradiation type; 300 W xenon lamp (*λ* > 420 nm); 1 h irradiation.

**Fig. 3:**
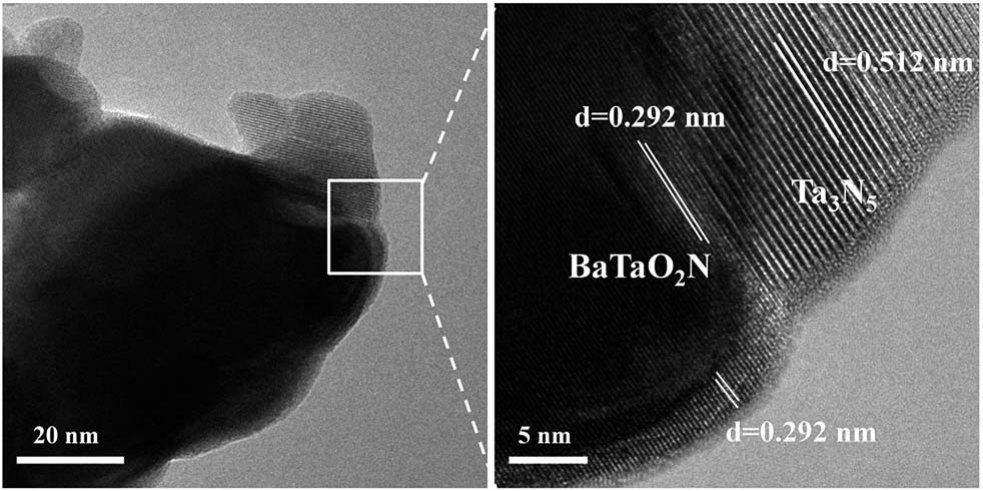
Representative TEM (left) and locally enlarged HRTEM (right) images of the chosen Ba(0.3)–Ta_3_N_5_ sample.

To further confirm the formation of the nanocomposite, we carried out a (HR)TEM characterization. Fig. 3 gives the representative images of the Ba(0.3)–Ta_3_N_5_ sample, in which the interface of the nanocomposite can be clearly observed. As shown in Fig. 3, the obvious lattice fringes indicate that the sample synthesized in this work is well-crystallized, in accordance with the XRD patterns (Fig. 1A). Based on the measurement of lattice distance, we can easily judge the BaTaO_2_N and Ta_3_N_5_ phases. Strikingly, the interfacial contact between BaTaO_2_N and Ta_3_N_5_ is very intimate, revealing the formation of the nanocomposite. The formation of the intimate interface should originate from the one-pot high temperature route and their similar Ta-based octahedron units. In this case, BaTaO_2_N is expected to be formed *in situ* on the surface of Ta_3_N_5_ during the one-pot nitridation process.

**Fig. 4:**
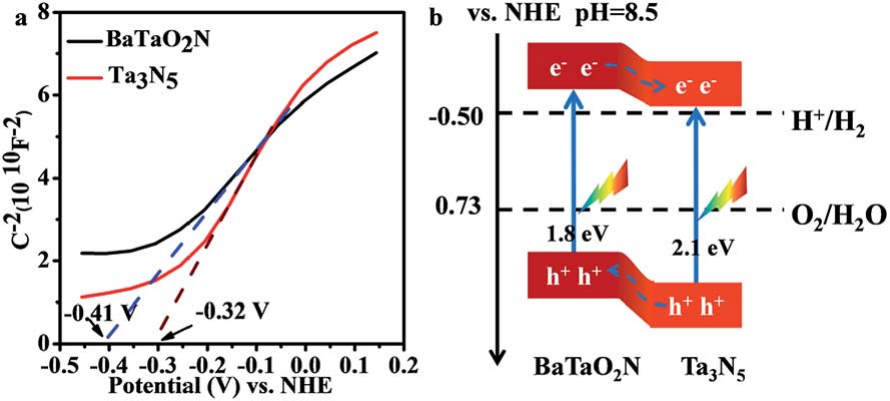
Band structure characterizations of the Ta_3_N_5_ and BaTaO_2_N samples. (a) Mott–Schottky plot for the Ta_3_N_5_ and BaTaO_2_N electrodes. Electrolyte: 0.5 M Na_2_SO_4_ solution (pH = 8.5, adjusted using NaOH). Frequency: 1000 Hz. (b) The relative band positions of the Ta_3_N_5_/BaTaO_2_N heterostructure.

The relative band positions of Ta_3_N_5_ and BaTaO_2_N were analysed by combining their Mott–Schottky (M–S) plots and UV-Vis results. In Fig. 4a, the flat band potentials of BaTaO_2_N and Ta_3_N_5_ were evaluated according to M–S measurement results to be *ca.* –0.41 V and –0.32 V *vs.* NHE, respectively. In consideration of the fact that the bottom of the conduction band (CB) for one n-type semiconductor is normally more negative by *ca.* 0.2 V than the flat band potential,^24,45,46^ the CB positions of the n-type Ta_3_N_5_ and BaTaO_2_N are estimated to be –0.52 eV and –0.61 eV, respectively. By combining their bandgaps achieved from the UV-Vis results (Fig. 1B), the relative band positions of BaTaO_2_N and Ta_3_N_5_ are then deduced and given in Fig. 4b. Accordingly, the nanocomposite exists as a type II heterostructure, where the excited electrons are expected to transfer from the conduction band of BaTaO_2_N to that of Ta_3_N_5_, while the photogenerated holes will transfer in an opposite way, leading to the spatial charge separation.

The photocatalytic H_2_ evolution rates on the pristine and modified Ta_3_N_5_ samples were examined using the deposited platinum nanoparticle as the reduction cocatalyst in the presence of CH_3_OH under visible light irradiation (*λ* > 420 nm). No reaction takes place in the dark, and H_2_ is evolved only under light irradiation. As given in the half reaction part of Table 1, the rate of H_2_ evolution undergoes an initial increase and subsequent decrease with the increasing molar ratio of Ba/Ta, and the optimal value of the Ba/Ta molar ratio is 0.3. Compared to the Ta_3_N_5_ (entry 1), BaTaO_2_N (entry 9) or Ta_3_N_5_/BaTaO_2_N (0.3)-mix (entry 10) sample, the H_2_ evolution rate on the Pt/Ba(0.3)–Ta_3_N_5_ photocatalyst is remarkably promoted. The typical time curve of H_2_ evolution on the Pt/Ba(0.3)–Ta_3_N_5_ sample is given in Fig. S3,† in which it is almost linearly increased in the experimental region, demonstrating its good photochemical stability. In addition, only a small amount of N_2_ (less than 1 μmol) was detected in the initial stage of irradiation. The dependence of the H_2_ evolution rate on the Pt/Ba(0.3)–Ta_3_N_5_ photocatalyst as a function of irradiation wavelength is well consistent with that of the UV-vis spectra (Fig. S4†), indicating that the H_2_ evolution process is driven by the incident light.

**Fig. 5:**
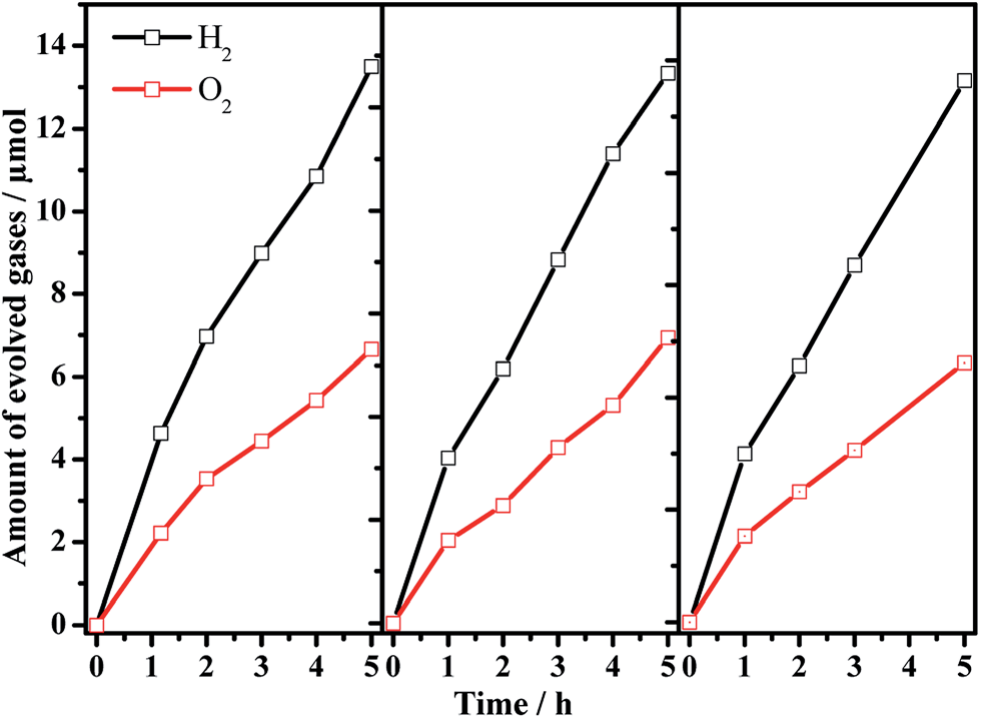
Multiple cycles of Z-scheme overall water splitting with 0.5 wt% Pt/Ba(0.3)–Ta_3_N_5_ and 0.45 wt% PtO_*x*_/WO_3_ as H_2_-evolving and O_2_-evolving photocatalysts, respectively. Reaction conditions: 50 mg of Pt/Ba(0.3)–Ta_3_N_5_ and 50 mg of PtO_*x*_/WO_3_; 100 mL of NaI aqueous solution (1.0 mM); 300 W xenon lamp (*λ* > 420 nm), top-irradiation.

Encouraged by the significantly enhanced H_2_ evolution rate, we tried to use the pristine or barium-modified Ta_3_N_5_ samples as H_2_-evolving photocatalysts to construct a Z-scheme overall water splitting system together with a PtO_*x*_/WO_3_ and IO_3_^–^/I^–^ pair as an O_2_-evoloving photocatalyst and redox mediator, respectively. As shown in the overall water splitting part of Table 1, when using pristine Ta_3_N_5_ as the H_2_-evolving photocatalyst (entry 1), no obvious H_2_ evolution is detected, demonstrating the infeasibility of Ta_3_N_5_ itself to drive the Z-scheme overall water splitting process. However, using the barium-modified Ta_3_N_5_ samples as H_2_-evolving photocatalysts (entries 2–8), overall water splitting with H_2_/O_2_ molar ratios of close to 2 : 1 is achieved, and the photocatalytic activity is dependent on the Ba/Ta molar ratio with an optimal value of *ca.* 0.3. The Z-scheme activities using the barium-modified samples as H_2_-evolving photocatalysts (entries 2–8) are all higher than those using BaTaO_2_N (entry 9) or the mixed sample (entry 10). The AQE was measured using the Ba(0.3)–Ta_3_N_5_ sample as the H_2_-evolving photocatalyst to be 0.1% at 420 nm. The activity trend is similar to the result of the photocatalytic proton reduction reaction, indicating that the overall water splitting performance is rate-determined by the H_2_-evolving side. In addition, the multiple cycles of time course curves further demonstrate its photochemical stability in the experimental region (Fig. 5). No obvious Ba^2+^ ion residue is observed in the centrifuged solution after reaction.

**Fig. 6:**
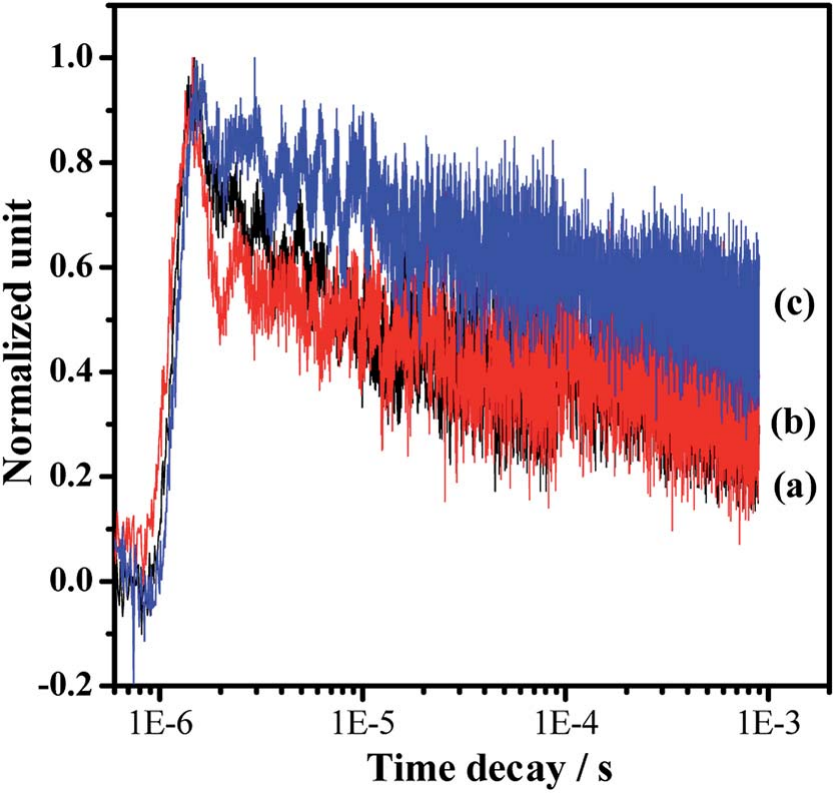
Normalized transient absorption profiles of the representative samples in a vacuum: (a) Pt/Ta_3_N_5_, (b) Pt/BaTaO_2_N and (c) Pt/Ba(0.3)–Ta_3_N_5_. The pulse laser at 355 nm was used to excite the samples for the IR tests. The cocatalyst of Pt with a loading amount of 0.5 wt% was deposited by impregnation and a subsequent H_2_ reduction method.

Photocatalytic overall water splitting commonly confronts huge challenges from both thermodynamic and kinetic aspects.^2,47^ In the past few decades, many candidate materials have satisfied the thermodynamics requirement, but overall water splitting on them is unfeasible because of the constraint of insufficient reaction kinetics in the H_2_ and/or O_2_-evolving side. Accordingly, developing strategies to address the water splitting reaction kinetics, which is greatly affected by the charge separation and surface catalytic process, is highly valuable. In this work, we adopt a simple one-pot nitridation approach with an ammonia flow (250 mL min^–1^) at high temperature (1223 K) to address the key issue of the charge separation *via* barium modification of Ta_3_N_5_. Based on our modification, not only is the defect density of Ta_3_N_5_ decreased, but also a Ta_3_N_5_/BaTaO_2_N heterostructure with intimate interfacial contact is formed for the promotion of spatial charge separation. Both of these are reasonably responsible for promoting photogenerated charge separation, contributing to the enhanced proton reduction performance as well as the feasible overall water splitting process. The promotion of charge separation is confirmed by comparing the time-resolved infrared spectra (TRIR) of the typical Ta_3_N_5_, BaTaO_2_N and Ba(0.3)–Ta_3_N_5_ samples (Fig. 6). The effective formation of the Ta_3_N_5_/BaTaO_2_N heterostructure probably originates from their similar structure units containing Ta-based octahedra. The decreased defect density of Ta_3_N_5_ originates from the part doping of Ba ions and the formation of BaTaO_2_N on the surface of Ta_3_N_5_ leading to surface passivation. It needs to be pointed out that with the increasing Ba/Ta molar ratio, the content of BaTaO_2_N with the highest defect density (see UV-Vis results in Fig. 1B) is enhanced, resulting in the increase of recombination centres, which is unfavourable for the photocatalytic H_2_ evolution process. As an integral factor of the heterostructure and the defect centres, the photocatalytic activity exhibits an initial increase and a subsequent decrease with the increasing molar ratio of Ba/Ta.

## Conclusions

In summary, a simple one-pot nitridation strategy is adopted for the barium modification of Ta_3_N_5_ photocatalyst to address its poor photogenerated carrier separation ability as well as H_2_-evolving activity. The one-pot nitridation route overcomes well the challenge of low thermal stability in air for (oxy)nitride-related photocatalysts. Based on this, barium ions are partially doped into Ta_3_N_5_ to inhibit the formation of defects, and the residue amount of barium ions will cause the *in situ* formation of BaTaO_2_N on the surface of Ta_3_N_5_ to create an intimate interface for the Ta_3_N_5_/BaTaO_2_N heterostructure. Both of the structures favour the enhancement of charge separation efficiency as well as the promotion of the H_2_-evolving rate. Finally, we successfully achieve a Z-scheme overall water splitting process under visible light irradiation using the Ba-modified Ta_3_N_5_ as a H_2_-evolving photocatalyst. The fabrication of the heterostructure *via* a one-pot route is expected to be extended into more (oxy)nitride systems for promoted solar energy conversion.
