# Supplementary material for: Achievement of visible-light-driven Z-scheme overall water splitting using barium-modified Ta3N5 as a H2-evolving photocatalyst
Source: Chem Sci. 2016 Aug 18;8(1):437–43. doi: 10.1039/c6sc02750d (PMC5365062; doi:10.1039/c6sc02750d)
Supplement: Supplementary file 1 [file SC-008-c6sc02750d-s001.pdf]

Electronic Supplementary Information

Achievement of Visible-light-driven Z-scheme Overall Water Splitting Using Barium-modified Ta_3N_5 as a H_2-evolving Photocatalyst

Yu Qi,[a,b] Shanshan Chen,[a] Mingrun Li,[a] Qian Ding,[a,b] Zheng Li,[a,b] Junyan Cui,[a,c] Beibei Dong,[a,b] Fuxiang Zhang,*[a] and Can Li*[a]

[a] State Key Laboratory of Catalysis, *i*ChEM, Dalian Institute of Chemical Physics, Chinese Academy of Sciences, Dalian National Laboratory for Clean Energy, Dalian, 116023, China
[b] University of Chinese Academy of Sciences, Beijing 100049, China
[c] Key Laboratory of Surface and Interface Chemistry of Jilin Province, College of Chemistry, Jilin University, Changchun 130021, China
E-mail: fxzhang@dicp.ac.cn, canli@dicp.ac.cn

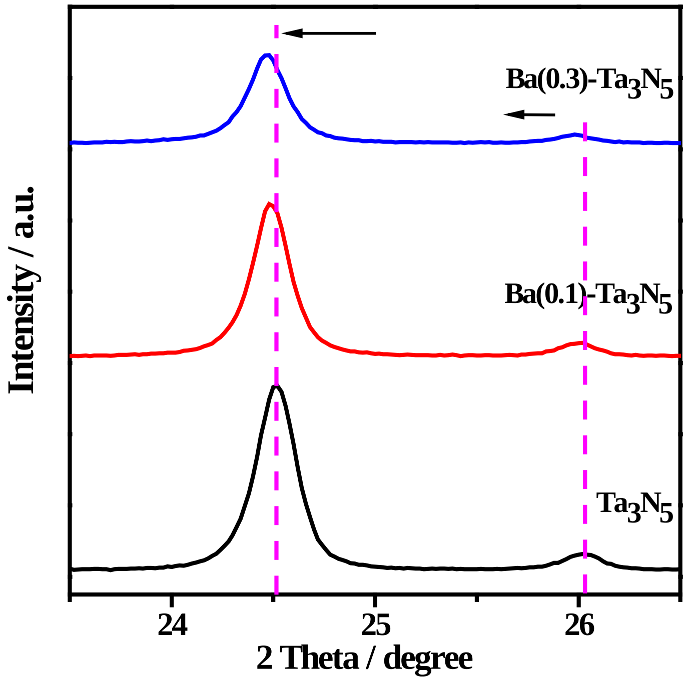

Figure S1. XRD patterns of several typical samples.

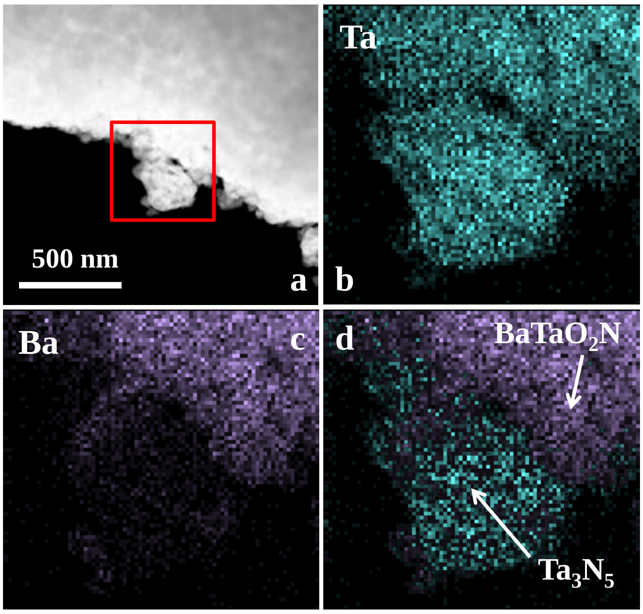

Figure S2. The elemental mappings of the mixture of Ta_3N_5 and $BaTaO_2N$: (a) TEM image, (b) Ta element, (c) Ba element, (d) simulated dispersion of Ta_3N_5 and $BaTaO_2N$.

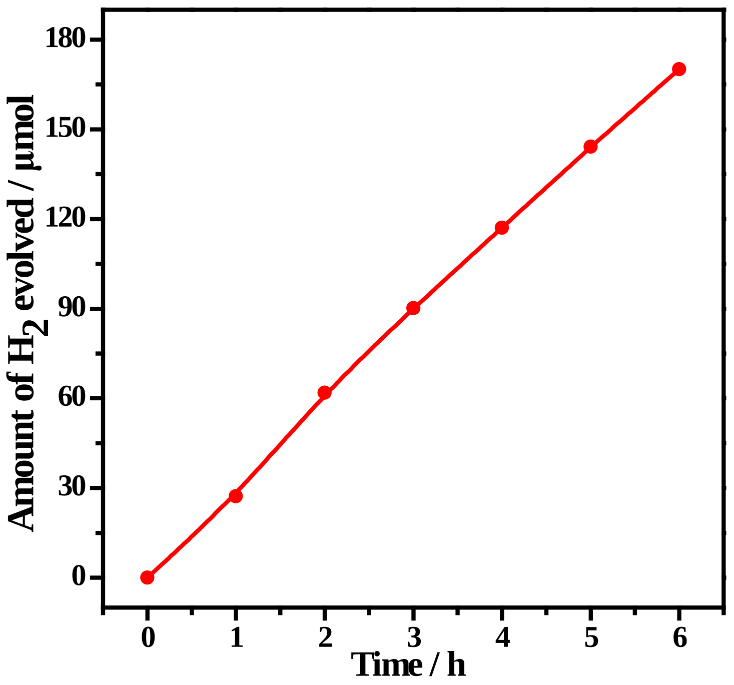

Figure S3. Time course of photocatalytic H_2 evolution on 0.5 *wt*% Pt/Ba(0.3)-Ta_3N_5 under visible light irradiation (λ > 420 nm). Reaction conditions: 0.15 g catalyst; 0.15 g La_2O_3; aqueous methanol solution (150 mL, 20 *vol*%); light source, 300 W xenon lamp, Pyrex top-irradiation type.

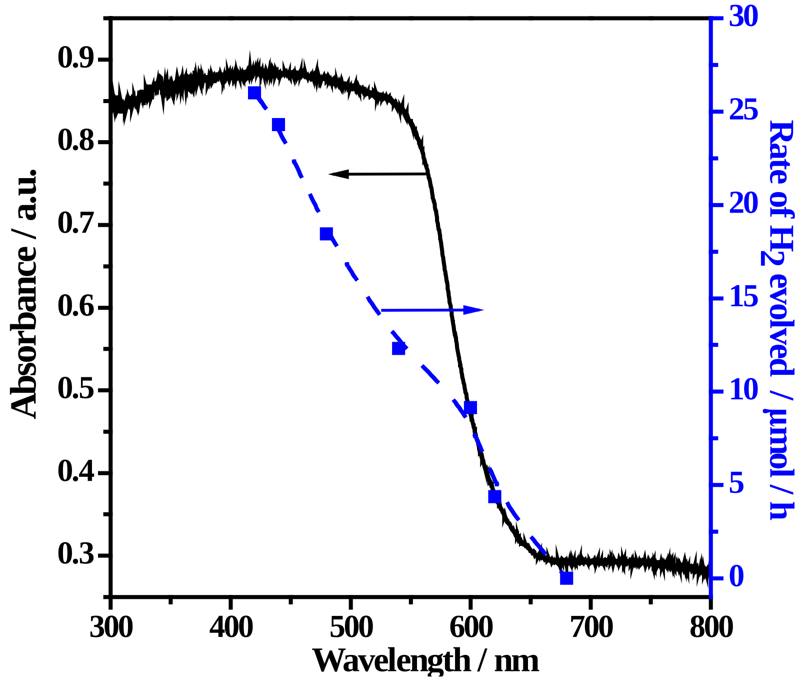

Figure S4. Dependence of the H_2 evolution rate on the cutoff wavelength of incident light (blue line) and the UV-vis DRS of Ba(0.3)-Ta_3N_5 sample (black line). Reaction conditions: 0.15 g 0.5 *wt*%Pt/Ba(0.3)-Ta_3N_5; 0.15 g La_2O_3; aqueous methanol solution (150 mL, 20 *vol*%); 300 W xenon lamp (λ > 420 nm); 1 h reaction time.
